# Interleukin-6 “Trans-Signaling” and Ischemic Vascular Disease: The Important Role of Soluble gp130

**DOI:** 10.1155/2017/1396398

**Published:** 2017-01-31

**Authors:** Mario Luca Morieri, Angelina Passaro, Giovanni Zuliani

**Affiliations:** ^1^Section of Internal and Cardiorespiratory Medicine, Medical Science Department, University of Ferrara, Ferrara, Italy; ^2^Section of Internal and Cardiorespiratory Medicine, Department of Morphology, Surgery, and Experimental Medicine, University of Ferrara, Ferrara, Italy

## Abstract

Inflammation plays a major role in the onset of cardiovascular disease (CVD). Interleukine-6 (IL-6) is a multifunctional cytokine involved both in the beneficial acute inflammatory response and in the detrimental chronic low-grade systemic inflammation. Large genetic human studies, using Mendelian randomization approaches, have clearly showed that IL-6 pathway is causally involved in the onset of myocardial infarction. At the same time, IL-6 pathway is divided into two arms: classic signaling (effective in hepatocytes and leukocytes) and trans-signaling (with ubiquitous activity). Trans-signaling is known to be inhibited by the circulating soluble glycoprotein 130 (sgp130). In animal and in vitro models, trans-signaling inhibition with sgp130 antibody clearly shows a beneficial effect on inflammatory disease and atherosclerosis. Conversely, epidemiological data report inconsistent results between sgp130 levels and CV risk factors as well as CV outcome. We have reviewed the literature to understand the role of sgp130 and to find the evidence in favor of or against a possible clinical application of sgp130 treatment in the prevention of cardiovascular disease.

## 1. Introduction

Cardiovascular disease (CVD) is one of the leading causes of morbidity and mortality all around the world. Over the last decades, the role of inflammation in atherosclerosis has been widely recognized and studied [1]. Identification of the detailed pathways that link inflammation to atherosclerosis and CVD provides an auspicious ground to find new possible therapeutic targets. Since the last century, plasma C-reactive protein (CRP) levels have attracted great attention, showing robust results as a marker of systemic inflammation associated with cardiovascular risk [2]. We have recently reported that also among elderly population low-grade systemic inflammation, as identified by hsCRP levels, was associated with increased CV risk [3]. Conversely, while hsCRP is clearly a reliable marker to identify subjects at higher CV risk, it does not seem to be an effector of the inflammation-driven atherogenesis. Genetic studies have addressed this issue with the use of Mendelian randomization approach [4]. In this type of analysis, several authors have argued against a causal association of CRP with coronary heart disease due to the lack of consistency between the effect of CRP genetic variants on CVD and CRP levels [4–6]. Plasma CRP is produced primarily in the liver, as a response to inflammatory stimulation by cytokines, such as Interleukine-6 (IL-6). As reported by different studies, IL-6 represents an upstream inflammatory cytokine that seems to be responsible for the chronic-inflammation-related atherogenesis [7, 8]. The causal role of this pathway has been nicely shown in studies involving the principles of Mendelian randomization [9, 10].

**Figure 1:**
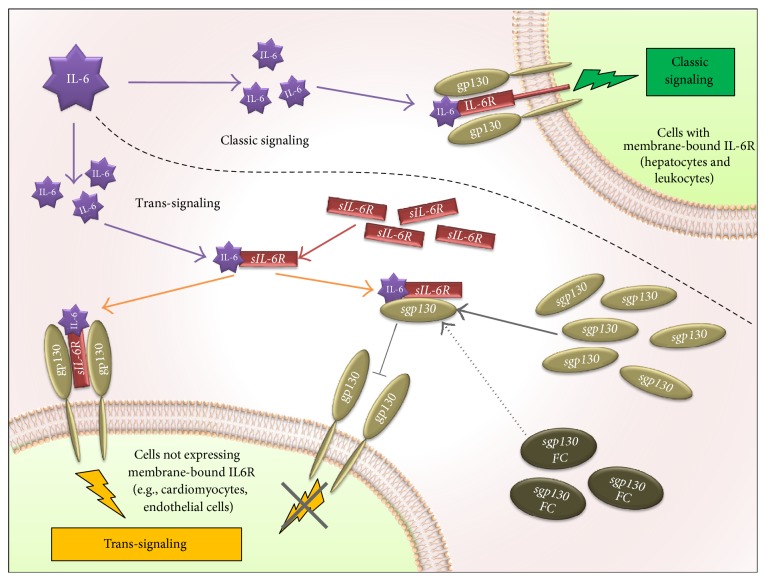
IL-6 signal transduction via classic and trans-signaling. The upper part of the figure depicts IL-6 signaling in cells expressing the membrane-bound receptor for IL-6 (IL-6R). In these cells (e.g., hepatocytes and several white blood cells), circulating IL-6 binds directly to IL-6R that forms a signaling complex with the membrane-bound glycoprotein 130 (gp130); this pathway is known as classic signaling. The bottom part depicts the IL-6 signaling in those cells that do not express the membrane-bound IL-6R. In these cells, membrane-bound gp130 (ubiquitously expressed) is activated by the circulating IL-6/sIL-6R complex (composed of IL-6 and the circulating soluble portion of IL-6R, sIL-6R). This pathway, known as trans-signaling, could be inhibited by the circulating soluble portion of gp130 (sgp130), which, by means of binding the circulating IL-6/sIL-6R complex, blocks the activation of the membrane-bound gp130. sgp130fc is a recombinant fusion protein of soluble gp130 and human IgG1 Fc that blocks IL-6 trans-signaling mimicking sgp130 functions.

As shown in Figure 1, IL-6 pathway could be differentiated into two axes, with different cell targets and divergent downstream effects. In the classic pathway, IL-6 binds the membrane-bound IL-6 receptor (IL-6R), located on the surface of hepatocytes and some leukocytes, and activates the IL-6 classic signaling transduction cascade with the homodimerization of the membrane-bound *β*-receptor glycoprotein 130 (gp130). In the “trans-signaling” axis, circulating IL-6 forms a heterodimer with the soluble form of IL-6 receptor, IL-6/sIL-6R, that could transduce a proinflammatory cascade in virtually any cell types through direct binding with membrane-bound gp130. The soluble form of the gp130 (sgp130) could instead inhibit the latter axis, through specific binding with the IL-6/sIL-6R heterodimer (interfering with its ability to bind the membrane gp130). Recently, new basic and clinical studies have highlighted the probable determinant role of the IL-6 trans-signaling pathway in the inflammatory-driven atherogenesis process. Our aim was to evaluate the current “state of the art” providing a comprehensive review of the relationship between CVD and IL-6 trans-signaling. Based on this review, we further speculate on the possible use of drugs targeting this pathway in the treatment of CVD.

## 2. IL-6 Classic and Trans-Signaling Effects

IL-6 is a cytokine with a multifactorial function and induces both pro- and anti-inflammatory responses [11]. It appears to have ubiquitous functions in several physiological and pathological processes [11, 12]. IL-6 has two different pathways for its induction of intracellular signaling: classic signaling (active primarily in hepatocytes and lymphocytes) and trans-signaling (with ubiquitous activity). The downstream effect of these signaling axes shows divergent functions [12]. Consistent data suggest that the classic signaling (through direct binding of membrane-bound IL-6R) is mainly responsible for the beneficial regenerative and antibacterial effects of IL-6 [13, 14], while the trans-signaling (through “IL-6/soluble- IL-6R heterodimer” bound to membrane-bound gp130) seems to account for the majority of the deleterious effect of IL-6 [15, 16]. While this simplistic view is nonexhaustive for the complex pathway of IL-6, it gives a glimpse of the reason why trans-signaling is considered so important. Furthermore, since sgp130 (the soluble form of gp130) is known to inhibit the IL-6/sIL-6R induced trans-signaling [17], it represents an ideal pharmacological target for IL-6 signaling.

Animal model and in vitro studies have reported the beneficial effect, in several inflammatory and degenerative disease models, of specific inhibition of trans-signaling with an fc-dimerized version of sgp130 (sgp130fc) [18–23] (as depicted in Figure 1). This process has been consistently reported also in cardiometabolic disease. Kraakman and colleagues showed that specific inhibition of trans-signaling, with sgp130fc protein, prevents the recruitment of macrophages in adipose tissue induced by high fat diet (ATM recruitment). On the contrary, in the same study, the complete blockade of IL-6 (both classic and trans-signaling) exacerbates obesity/induced weight gain, liver steatosis, or insulin resistance [24].

Schuett et al. have shown the protective effect of sgp130 in an animal model of atherosclerosis. Treatment with sgp130fc attenuates the atherosclerotic lesion progression in LDL^−/−^ mice by decreasing endothelial activation, smooth muscle cell infiltration, and monocyte recruitment [25]. Furthermore, these authors assessed the therapeutic relevance of sgp130fc in a model of preexisting atherosclerosis, showing a reduction of thoracoabdominal lipid deposition and of aortic root lesion size with this treatment. In addition, Schuett et al. confirmed that sgp130fc did not influence hepatic effects of IL-6 (suggesting preserved IL-6 classic signaling). This specific and trans-signaling-targeted effect of the sgp130fc is of great relevance since other studies have shown that overall IL-6 signaling has also a beneficial cardiometabolic function, and complete blockade of IL-6 could be counterproductive [26–28].

## 3. Trans-Signaling and Cardiovascular Disease in Human

### 3.1. Epidemiological Studies on sgp130 Levels

While animal models provided consistent results for the beneficial consequences of the sgp130fc-induced blockade of the IL-6 trans-signaling, in human studies, the association of sgp130 levels and cardiovascular disease appears less straightforward. Indeed, while some studies reported an inverse association between sgp130 levels and CVD, others reported null or positive association.

Schuett et al. confirmed the translational relevance of the beneficial effect of sgp130fc treatment showing that sgp130 levels were lower among 50 patients with coronary artery disease (CAD) as compared to controls [25]. Furthermore, among patients with CAD, sgp130 levels were inversely associated with extension of disease. Anderson et al., in a similar sample size, found that sgp130 had an inverse association with previous myocardial infarction (MI), although there were no differences between patients with acute MI and CAD. Interestingly, sgp130 levels had a positive correlation with the peak of troponin I [29]. In a much larger population-based case-control study, involving 664 cases and 1062 controls, very high levels of sgp130 were associated with a 30% reduction in the incidence of myocardial infarction (OR: 0.7; 95% CI: 0.5–0.9) [30].

On the contrary, different studies showed a negative prognostic value of sgp130 among those patients with a history of MI [31] and in particular among subjects with heart failure (HF) [32, 33]. Indeed, serum levels of sgp130 were reported to be higher among patients suffering from chronic heart failure [34, 35] and, most of all, as reported by Askevold et al., to be associated with CV and total mortality in elderly patients with HF of ischemic cause. In this study, subjects with high levels of sgp130 (those in the 5th quintile versus all the others) had a significant 38% increase in CV mortality, a 47% increase in all-cause mortality, and an 85% increase in death from worsening of HF [32].

A possible explanation for these apparently counterintuitive results is that, in the context of chronic ischemic disease and vascular remodeling, higher sgp130 levels are representative of a compensatory response to higher activation of the IL-6 signaling, with increased gp130 expression. In support of this hypothesis, Inta et al. reported that sgp130 levels correlated with blood pressure and carotid intima-media thickness in stroke patients and that these increased levels may reflect the vascular remodeling response to arterial hypertension, as suggested by the increased gp130 mRNA expression in the aortic wall of spontaneous hypertensive rats [36]. Furthermore, we have recently found that also among community dwelling elderly individuals there was a significant association between sgp130 levels and metabolic syndrome; nevertheless, this association seemed to be mediated by insulin resistance [37].

Thus, it is possible that in these groups of patients higher sgp130 represents a marker of higher fragility more than being a cause of adverse outcome.

### 3.2. Genetic Variants in IL-6R and gp130 and Cardiovascular Disease

By using the principle of the Mendelian randomization, it is possible to address the issue of causality. The general principle of these studies is that lifelong genetically determined exposure to a marker of CV risk factor should induce higher prevalence of CVD only if this risk factor is a causal mediator of the disease.

In two independent large-scale human genetic studies, a functional genetic variant (Asp358Ala) located in the gene coding the IL-6R has been shown to be associated with lower coronary heart disease (CHD) [9, 10]. This nonsynonymous variant (358Ala), located in the cleavage site of IL-6R, confers increased proteolytic conversion rates by ADAM proteases (ADAM10 and ADAM17) [38], resulting in higher circulating levels of soluble IL-6 receptor and lower downstream transduction of IL-6 signals. As a consequence, carriers of the alternative allele, those with lower risk of CHD, have a 2-fold increase in the circulating levels of soluble IL-6R and reduced downstream IL-6 signaling as demonstrated by the lower levels of hsCRP and fibrinogen. A further consequence of this functional variant is positive feedback with paradoxical increase in IL-6 levels. Thus, from these studies, it is possible to confirm the causal association between IL-6 signaling and CHD but it is not possible to depict whether the reduced transduction of the IL-6 signal involved only the classic signaling (as suggested by the hsCRP and fibrinogen lower levels) and/or the trans-signaling too.

Given the higher levels of IL-6 and sIL-6R associated with the 358Ala variants, one would expect that trans-signaling should be increased as well. Conversely, the opposite scenario is also possible; indeed, it must be considered that the increase in levels of the soluble IL-6R could potentiate the antagonistic activity of sgp130 on IL-6 response [39]. As mentioned also by Scheller and Rose-John [40], the decoy receptor sgp130 has a much higher concentration (≈200 *μ*g/L) than soluble IL-6R (≈50 *μ*g/L) and IL-6 (≈2 ng/L) [12, 37]. Thus, it is probable that among carriers of the alternative allele (with 358Ala) the higher soluble IL-6R levels improve the buffer activity of sgp130 with reduced ubiquitous IL-6 trans-signaling (as well as the reduced classic signaling in hepatocytes and leukocytes [41]).

### 3.3. gp130 Genetic Variants and Cardiovascular Disease

Further evidence for the role of sgp130 in the onset of cardiovascular disease has been reported in studies on genetic variants located in the gene coding gp130* (IL6ST)*.

Lucthefeld et al., in a haplotype-based analysis, identified that genetic variability in the* IL6ST* gene was associated with CAD and MI in two independent populations [42]. Interestingly, in this study, a highly significant association was detected with the atherosclerosis of the ostium of the coronary arteries, which has an important clinical relevance for the coronary flow.

Bernick et al. then analyzed one of the nonsynonymous single nucleotide polymorphisms studied in this paper (Gly148Arg, rs3729960) [43]. This functional SNP is known to change the stability of the glycoprotein and influence the responsiveness to IL-6, as shown by the slightly reduced transduction of the signals associated with the 148Arg allele [43]. Most interestingly, this variant was associated with a 46% decreased risk of myocardial infarction, confirming the previous report by Lucthefeld et al. Recently, Wonnerth et al. have shown that carriers of the 148Arg allele had higher circulating levels of soluble gp130 (sgp130); interestingly, they were able to replicate these results in two different cohorts [44]. Even though this data suggests that the lower risk of MI in 148Arg carriers could be mediated by higher sgp130 circulating levels, this could not be proven at this point. Indeed, cells transfected with the 148Arg allele showed lower activity of the membrane-bound receptor, and furthermore this amino acid change is located in the cytokine binding site; thus, it is not possible to exclude an altered interaction with the IL-6/sIL-6R heterodimer.

Finally, it is important to notice that this and other variants in the IL6ST gene have been associated with increased prevalence of metabolic syndrome and higher BMI [44, 45]. These results confirmed the complexity of this pathway in the onset of cardiometabolic disease; nevertheless, animal models suggest that only the complete blockade of IL-6 (both classic and trans-signaling) exacerbates obesity and insulin resistance [24], while this effect was not present in specific inhibition of the trans-signaling.

## 4. Conclusion

Current IL-6 blockade treatments, used in specific inflammatory diseases, such as rheumatoid arthritis, are nonspecific (targeting both IL-6 classic and trans-signaling) and are associated with increased infection and metabolic disturbances. Development of new treatments (e.g., sgp130fc) aiming at specific inhibition of IL-6 trans-signaling seems to be a promising avenue also for the treatment and prevention of cardiovascular disease. Nonetheless, given the complexity of the IL-6 cascade, further studies to confirm this hypothesis are warranted. Specifically, human genetic studies, conducted in large and different cohorts, could provide interesting validation of this hypothesis; furthermore, these studies could identify specific subjects who may benefit more from this possible treatment.
